# Genetic and Environmental Determinants of Beef Quality—A Review

**DOI:** 10.3389/fvets.2022.819605

**Published:** 2022-02-24

**Authors:** Tomasz Sakowski, Grzegorz Grodkowski, Marcin Gołebiewski, Jan Slósarz, Piotr Kostusiak, Paweł Solarczyk, Kamila Puppel

**Affiliations:** ^1^Department of Biotechnology and Nutrigenomics, Institute of Genetics and Animal Biotechnology, Jastrzebiec, Poland; ^2^Department of Animal Breeding, Institute of Animal Sciences, Warsaw University of Life Sciences, Warsaw, Poland

**Keywords:** red meat, quality, beef, fatty acid composition, breed

## Abstract

The flavor, quality, and composition of beef changes with the cattle diet regimen. The quality of meat varies, and that variability is determined by both individual and environmental factors: age, breed, live weight, fatness degree, plane of nutrition, and concentrate/roughage ratio. The strategy for the rearing and feeding of cattle for slaughter should therefore aim at reducing the saturated fatty acid content and increasing the polyunsaturated fatty acid and monounsaturated fatty acid levels. Many diseases in humans, like atherosclerosis and cardiovascular diseases, are associated with dietary fat, and their development process could take a year, the results of which can be a shorter life and its lower quality. The objective of this review was to describe the factors affecting the meat quality and fatty acid profile of the intramuscular fat of European cattle fed various diets.

## Introduction

According to Directive 2001/101/EC^1^, products containing more connective tissue and fat should be labeled as “mechanically separated meat.” In the case of beef, the term “good quality of meat” means that the meat is free from all quality defects and that it is originated from healthy animals. All changes occurred correctly at an appropriate pace both in the living animals and in the slaughtered carcasses. It also means that the meat has been cut from the carcass and delivered for consumption or processing after reaching a sufficient degree of ripeness. Listrad et al. (1) pointed out that the quality of the meat can be described by 4 terms: security, healthiness (nutritional quality), sensory quality, and serviceability.

Meat is a mixture of several compounds, but its basic ingredients include water (70–76%), protein (18–23%), nitrogen compounds (1–3%), carbohydrates (0.5–2%), muscle fat (0.7–10%), and mineral components (0.5–2%). The development of the basic chemical composition of meat and thus the development of its nutritional quality are influenced by many factors, both genetic and environmental (1–5).

Water is the predominant component of beef, accounting for ~70–76%. It occurs in a bound form (indistinguishable by centrifugation and pressure) and in the free form. Differences in water concentration in muscle tissue result primarily from the different content of intramuscular fat. In addition, the age of the animals also shapes the level of this parameter, because the water content decreases with the age of the animals (6).

The total protein content of meat is in the range of 15–23% (7–9). It is believed that white meat is healthier than red meat. However, when assessing the healthiness of meat, protein content in particular types of meat is important, but not the most important factor. Beef is characterized by a better amino acid profile compared to other meats. It contains significantly more branched-chain amino acids, including valine, leucine, and isoleucine. Beef is also a rich source of amino acids that pass through the blood/cerebrospinal fluid barrier (10).

Beef meat is characterized by moderate and quite varied fat content, ranging between 0.6 and 23.3% weight of tissue (11). Fat content changes with age and intensity of nutrition (12). In late-maturing (large caliber) animals, the growth phase, which is characterized by high fat deposition, is shifted over time, so it is possible to link it to high body weight without worrying about lowering the carcass quality. Individual breeds differ from each other in the composition of intramuscular fat as well as the ratio between the different types of fibers. Late-maturing breeds such as Belgian Blue, Limousine, and Blonde d'Aquitaine are characterized by a better muscle tone and less fat (13) compared to those achieved by early-maturing breeds such as Angus. In addition, studies have shown a relationship of single-nucleotide polymorphism in candidate genes (calpastatin) with tenderness (14).

A unique feature of meat is its hydrophilicity, that is, the ability to bind and add water. Water absorption is a factor shaping the organoleptic characteristics of meat. During the ripening process, the ability of meat to bind water increases as a result of loosening the muscle protein grid (15).

The color of meat is one of the meat characteristics that are firstly evaluated by the consumer and, on this basis, shapes the image of the culinary applications of the meat (16). The color is a factor of the age of the animal, its nutrition, the conditions of keeping the animals before slaughtering, and the conditions of ripening. Meat becomes darker with age, changing color from red to dark red (15). Fresh beef should have a bright red color, which is mainly formed by the concentration and form of myoglobin and, to a lesser extent, by hemoglobin (the content in meat ranges from 6 to 16% of total heme coloration and mainly depends on the anatomical origin of the meat) and cytochrome c (17). Myoglobin (Mb) is a water-soluble hemoprotein that occurs in skeletal and cardiac muscles. Myoglobin concentration is determined by the species, breed, age, sex, feeding system, and physical activity of the animal. Cow muscles contain more myoglobin than heifers, bulls, or wolves. Myoglobin is at the level of 1–3 mg/g in muscles in calves, 6–10 mg/g in young cattle for slaughter, and 16–20 mg/g in cows for slaughter (18). Myoglobin occurs in three forms of redox: deoxymyoglobin (DMb), oxymyoglobin, and metmyoglobin (MMb). In turn, the redox form depends on the presence of a ligand connected to the iron atom of hem and on the value of iron (Fe^2+^, Fe^3+^). Deoxymyoglobin is a purple-red pigment which retains its form in fresh meat only at a low partial oxygen pressure of <1.4 mmHg. In the presence of oxygen, DMb is spontaneously oxidized to oxymyoglobin (19). When both ferrous myoglobin derivatives are oxidized to iron (Fe^3+^), it is transformed into metmyoglobin. MMb is the most undesirable form of heme pigment in muscles both in the vital period and in the post-mortem period (20). The formation of MMb is maximal when the partial pressure of oxygen is about 4 mmHg (18). Metmyoglobin is reduced by a complex of MMb reductase, cytochrome b5, and NADH. It should be noted that higher physical activity in the vital period increases the reductase activity (19). The stabilization of meat color in the post-mortem period depends on the activity of MMb reductase, which is the highest in the temperature range of 30–37°C. Both the higher antioxidant potential of the meat and its storage in the dark increase its activity (18). In addition, the muscles differ in color stability, where the highest stability is attributed to m. longissimus dorsi, followed by m. semimembranosus and m. gluteus medium, and the lowest to m. psoas major. The structure of the muscle proteins is also important and is a function of pH values; a dark color is accompanied by higher pH values (18, 21).

The pH value of the meat reflects the changes that occur after slaughter, i.e., the degree of maturity of the meat and its durability and usefulness. The lactic acid formed during anaerobic glycogenolysis acidifies the environment, and this process may last until the glycogen stores are exhausted or glycolytic enzymes are inactivated by a low pH. During these transformations, the pH of meat decreases from 7.0 to 5.5–5.6. The glycogen content significantly determines the final pH of the meat and the degree of protein proteolysis. In addition, it significantly affects water absorption, fat emulsifiability, tenderness and juiciness, taste, and smell. If the pH value is lowered too quickly, meat with a watery structure (piles, soft, exudative meat) will occur (18). However, the slower rate of glycolysis and changes in pH is the reason for the occurrence of dark, firm, dry (DFD) meat. Post-mortem proteolysis during meat maturation is a function of pH and temperature (16). The degree of acidification of the meat mainly affects the extent of the proteolysis, while the temperature of the environment influences its rate. Transport and pre-slaughter stress are factors that significantly increase the glycogen levels in the muscles after slaughter. At pH24 (24 h after slaughter), values above 6.0 are considered to be typical of DFD meat. This meat has a limited shelf-life (it may spoil after 7 days of refrigerated storage), is susceptible to bacterial spoilage, and is not suitable for the production of durable products (18). In addition, the dark, unnatural color negatively affects the appearance of the meat. Too advanced processes of meat maturation accompanied by the multiplication of microflora lead to its rotting decomposition—such meat is not fit for consumption. The characteristic features of this process are the appearance of stickiness and mucus on the surface of the meat, a change in smell (the release of unpleasant gases with a scent of hydrogen sulfide and ammonia), a visible change in color to dark red with a greenish or yellow tinge, and a meat pH exceeding 6.5 (16–18). Additionally, Rutherford et al. (22) reported that the rumen temperature can be used as a predictor of meat quality. Bulls with a greater rumen temperature during the pre-slaughter phase produced meat with a significantly higher pH_ult_. The flavor, quality, and composition of beef changes with cattle diet regimen (2). The types of forage fed to cattle affect both the carcass characteristics and gains. Beef quality, including its fatty acid composition, has recently been the focus of the interest of many researchers and customers. The genetic variability in beef quality has been linked to differences between lines or breeds, variations due to the crossing of breeds, and variations between animals (23). Differences between many breeds of cattle have been reported for Red Angus and Simmental steers (24), Aberdeen Angus, Belgian Blue, and Limousine bulls (25) and for different double-muscle genotype bulls (26).

Structural changes in the connective tissue are associated with the activity of cathepsin enzymes (21). Calpaine activity is responsible for the alteration of the proteolytic cytoskeletal and regulatory proteins of myofiber (27). In the skeletal muscle of the animals, 3 major types of calpains have been identified: m-calpain, μ-calpain, and calpaina 3; μ-calpain activity decreases sharply in the first days after slaughter, while m-calpain activity is stable (28). The National Institutes of Health issued detailed recommendations on the intake of long-chain n-3 fatty acids, recommending that at least 650 mg/day C20:5 n-3 and C22:6 n-3, 2.22 g/day C18:3 n-3, and 4.44 g/day C18:2 n-6 should help reduce the risk of cardiovascular diseases (29). Although seafood is the main source of polyunsaturated fatty acid (PUFA) n-3 fatty acids in the human diet, studies clearly indicate that red meat can also be an excellent source.

It is the intent of this review to synthesize and summarize the currently available information about beef quality as well as to discuss the interpretation of the results.

## Dietetic Properties of Beef

The share of individual fatty acid families in bovine intramuscular fat is as follows: 38–44% are saturated fatty acids (SFA), 46% are monounsaturated fatty acids (MUFA), and 10% are PUFA (18). Studies have shown that C12:0, C14:0, and C16:0 have atherogenic properties, while C14:0, C16:0, and C18:0 have thrombogenic properties (30). MUFA as well as fatty acids from the families PUFA n-3 and PUFA n-6 have anti-atherogenic and antithrombogenic effects. The quality of meat varies, and that variability is determined by both individual and environmental factors: age, live weight, fatness degree, plane of nutrition, and concentrate/roughage ratio (18, 29, 31). Barton et al. (32) revealed that supplementation of sunflower seed increased the proportions of C18:2 n-6 and C18:2 *cis-*9 *trans-*11 and PUFA/SFA ratio and decreased the fatty acid atherogenicity in meat lipids. Pasture-finished cattle produce beef with a greater concentration of PUFA n-3 fatty acids than concentrate-fed cattle (33–35).

Crude glycerin can be used as a long-term substitute for barley meal in concentrations of up to 10% of dry matter in the diets of finishing bulls (36). The different quantities of glycerin in ruminants might be either converted to volatile fatty acids, especially butyrate and propionate at the expense of acetate, or directly absorbed from the digestive system and act as a gluconeogenesis precursor in the liver (37, 38). Glycerin supplementation may also improve forage digestibility and increase the production of microbial proteins in the rumen in a dose-dependent manner (39). Glycerin addition in ruminant diet has also been estimated in several studies with cattle (40–42), but the results for carcass characteristics and growth performance were inconclusive and ambiguous. The study of Barton et al. (36) showed that the partial replacement of barley with crude glycerine did not have a significant influence on the feed conversion ratio and daily gain of bulls. Similar to these results, Mach et al. (41) concluded that glycerine is a good energy ingredient replacement in the finishing diet of bulls, with no negative impact on feed efficiency and daily gains. Positive effects on feed efficiency and daily gains have been noted when dietary glycerin supplementation in steer and heifer finishing diets was included at <10% of dry matter (40, 42). Conversely, reduced feed efficiency and daily gains were noted when a diet containing 16% glycerin was used (42). Dietary glycerin supplementation did not change the carcass composition, slaughter characteristic, and chemical composition of musculus longissimus lumborum (MLL). However, it is noteworthy that all the fatness characteristics (internal fats, carcass separable fat, carcass fatness score, fat thickness on MLL, and petroleum ether extract of MLL) were numerically higher in glycerin-fed cattle (36).

The formation of the fatty acid profile is also related to the type of muscle. Studies have shown that the SFA concentration in femorsis biceps is more than 3 times higher than in semi-membranous biceps. When analyzing the MUFA and PUFA contents, we can also see the advantage of biceps femorsis over semimembranous biceps (31).

## Influence of Breed on Beef and Carcass Properties

Breed differences in the muscle lipid fatty acid profile are often affected by the intramuscular fat content (due to differences in the fatty acid composition of the major muscle lipid fractions) (12, 16). Differences in the fatty acid composition of crossbred cattle were determined by genetic differences, rather than by differences in the content of intramuscular fat (43). Iwanowska and Pospiech (44) revealed that variations in cattle slaughter value can be as high between breeds as within a single breed; they found that the culinary meat amount obtained from carcasses may be increased by a modification in the carcass cutting system.

The musculature of an animal is influenced by many genes, one of which is the gene coding myostatin, whose polymorphism is associated with the occurrence of a double-muscled phenotype in Belgian Blue and Piemontese cattle. The Piemontese breed has a deletion of 11 nucleotides in exon 3 of the gene located on chromosome 2. This mutation caused the loss of 3 amino acids (275–277) in the polypeptide protein chain. Exon 3 has an open reading frame; the deletion caused it to move and create a stop codon after 287 amino acids. This led to a shortening of the protein chain and thus a loss of protein function (45). The relative increase in the number of fibers is observed in early pregnancy (46), with the results in the calf having almost twice as many muscle fibers at the time of birth. Belgian Blue animals have an increased ability to convert feed into lean muscle and produce a higher percentage of the most desirable cuts of meat. These animals have less bone, less fat, and on average 20% more muscle compared with double-muscled (DBM) and normal Belgian Blue bulls, and it was found that the meat of DBM bulls contained about 3 times more PUFA content (27.5 vs. 11.3 g/100 g of FA) compared with normal animals (47).

The dietary and healthy benefits to humans are determined by the long-chain PUFA traits due to their anti-atherogenic, anti-inflammatory, and antithrombotic effects. Meat is one of the most nourishing dietary sources (48). The n-6/n-3 ratio is considered as a risk factor in coronary heart disease and cancer disease when it is higher than 4 (49). This indicator was significantly lower in the LCS (based on lucerne silage and legume–cereal mixture silage) bulls and well below the recommended maximum (34). A number of reports showed variations in beef cattle performance and carcass characteristics under similar production conditions due to breed effects in crossbreeding experiments (50–53). Barton et al. (5) evaluated the effects of breed on live weight gain, carcass composition, and slaughter characteristics, comparing these with those of Aberdeen Angus, Charolais, Hereford, and Simmental bulls. The target slaughter live weights were fixed at 550 kg for earlier-maturing breeds, i.e., Aberdeen Angus and Hereford, and at 630 kg for later-maturing breeds, i.e., Charolaise and Simmental. Charolaise and Simmental gained faster than Aberdeen Angus, while Hereford was intermediate. More valuable cuts were in Charolaise and Simmental. Hereford breed was characterized by the highest separable fat percentage. The thinnest subcutaneous fat over m. longissimus lumborumet thoracis was recorded in Charolaise and Simmental than in Aberdeen Angus and Hereford. The results of the experiment showed that earlier-maturing bulls had a lower live weight and produced more fat and less percentage of meat from high-priced cuts in comparison with later-maturing breeds.

Nogalski et al. (54) defined the impact of genotype and carcass conformation class on the slaughter quality of 200 young bulls. In this group were 108 crossbred bulls and 92 Holstein-Friesians (HF). They were slaughtered at the age of 21–22 months. The results of the classification placed 61.11% of crossbred beef bulls in R class (in EUROP system), and 56.53% of HF bulls were classified as O. Using the same conformation classes, the HF bulls were characterized by a lower slaughter quality than the crossbred beef bulls which had a higher content of fat by 0.42% and also better fatty acid composition of meat. The carcasses from too young cattle were characterized by a lower content of muscles, only slight marbling, and poor subcutaneous fat cover. According to Kaczmarek (55), early-maturing breeds like Hereford and Aberdeen Angus tend to deposit fat earlier and are intensively feed with concentrates, which causes their carcasses to be fatter. The late-maturing breeds, like Chianina, Charolaise, Limousine, or Piemontese, manifest a higher tendency to accumulate protein rather than fat. Those breeds are predisposed for intensive fattening due to their impressive daily weight gains. On the other hand, Nogalski et al. (34) concluded that crossbred animals had an advantage over HF bulls as exposed by the higher content of functional fatty acids in meat fat.

The physiological groups have a strong influence on the composition of carcass tissues (56). Heifers reach the finishing phase before steers, who, in turn, reach the finishing phase before bulls (57). Pogorzelska-Przybyłek et al. (58) reported that, in semi-intensive production systems, steers performed better than bulls, and HF × HH crosses were more suitable than HF × Limousin and HF × Charolais crosses. The quality of carcasses is influenced by two important factors, e.g., final body weight and age of the animals. Based on the slaughter value, Nogalski et al. (54) determined the most efficient finishing weight of young Polish Holstein Friesian × Limousine crossbred bulls and steers. Upon comparing the slaughter results of bulls and steers, it was shown that bulls have a better slaughter value, 1.07–2.60% higher percentage of carcass dressing, lower carcass fatness, and higher carcass conformation. On the other hand, Sharman et al. (59) showed that a moderate level of energy intake and lower sensitivity to changes in dietary protein levels weigh in favor of steers. Additionally, Blanco et al. (60) reported that fattening steers and especially heifers can lead to improved fat-related meat quality traits in lean breeds.

The slaughter performance and fattening of Polish Red Cattle bulls were investigated by Łappa et al. (61). They reported that the carcasses of animals 12 months of age contained 68.12% meat and 13.25% fat, while 15-month-old animals had 66.61% meat and 15.12% fat, respectively. Nahlik (62) reported that the carcasses of 15-month-old Polish Red bulls reached 71.01% meat and 12.24% fat. Oprzadek et al. (63) reported newer results for tissue composition of 12-month-old Polish Red bull carcasses which had 71.91% meat and 9.76% fat. The analysis of the Polish Red Cattle slaughter value shows similar traits to the Limousine breed (amount and quality of meat) and that it strongly exceeds that of the Hereford breed. Additionally, Pogorzelska-Przybyłek et al. (58) reported that dairy–beef crosses should be slaughtered at 21 months of age to improve the carcass quality.

The study carried out by Daszkiewicz and Wajda (64) showed that the dressing percentages of Black and White breed and Limousine breed were respectively, 50.22 and 61.84%. Monsón et al. (65) reported a similar result (61.40%) for the Limousine breed. Both results of Daszkiewicz and Wajda (66) and Monsón et al. (65) have been confirmed by Oprzadek et al. (63) in a study on 12-month-old bulls. The dressing percentage of Limousine was higher (59.25%) than the values obtained for Black and White (50.94%) and Hereford breeds (54.92%). Miciński et al. (67) reported 63.86% dressing percentage of the Limousine breed and 55.30% of the Hereford breed. As shown by Malau-Aduli et al. (68), in the adipose tissue of Limousine and Jersey cattle, the total MUFA content tended to increase with age.

Many researchers provide that the best moment to terminate the fattening of a herd is when the animals attain the so-called slaughtering maturity, i.e., best musculature and carcass tissue composition and developed culinary elements (66, 69). Feeds rich in proteins should be used during the most intensive muscle tissue development. It is not recommended to slaughter animals too early before they attain appropriate slaughter maturity.

## Influence of Feeding System on Beef and Carcass Properties

Differences in carcass composition between animals fed different diets can be attributed to the diet composition or the effect of growth rate. Bulls and steers fed concentrates, forage *ad libitum*, or finished on concentrates after grazing were slaughtered at a similar weight and had a similar dressing percentage and degree of fat cover (16). Garcia et al. (70) reported total CLA values in the longissimus dorsi muscle of 5.8 vs. 3.1 mg/g FAs in steers fed on pasture supplemented with cracked corn grain (1% live weight) compared to a corn-based concentrate with alfalfa hay. Additionally, Rutherford et al. (71) reported that a production system including a grazing period within bull beef production may be a more sustainable approach to producing Holstein bulls.

Other factors, such as nutrition, have also been found to influence the meat quality due to their regulatory effect on biological processes in muscle and on fat deposition (29, 32, 72). Various feeding strategies are often used to increase the content of PUFA n-3 fatty acid and to improve beef intramuscular PUFA n-6/PUFA n-3 ratio (2, 4, 73–77). The strategy for the rearing and feeding of cattle for slaughter should therefore aim at reducing the SFA content and increasing the PUFA and MUFA levels (18, 30). Cattle forage typically contain 1–4% lipids, mostly PUFA, including α-linoleic and acid linoleic acid (29). Fredriksson-Eriksson and Pickova (31) reported that a higher α-linoleic concentration in meat from pasture-fed bulls can be enhanced by its association with the thylakoid membranes in chloroplasts that can protect against ruminal biohydrogenation. Ground grass-fed beef had a greater concentration of C18:2 *cis*-9 *trans*-11 and C18:1 *trans*-11 (29, 31). French et al. (78) reported that decreasing the proportion of concentrate in the diet, which effectively increased grass intake, caused a linear decrease in the concentration of SFA and in the n-6/n-3 ratio and a linear increase in the PUFA/SFA ratio.

Replacement maize silage with alfalfa silage and legume–cereal mixture silage conspicuously increased the C18:3n-3 dietary intake (32). Nogalski et al. (34) reported that the low proportion of PUFA in the FA profile could be related to the age of bulls at slaughter (21–22 months). The PUFA content of intramuscular fat in m. longissimus dorsi decreases with age, reaching 25.5% at 7 months, 18.4% at 14 months, and 13.6% at 19 months (79). The recommended PUFA n-6/PUFA n-3 ratio by the FAO and WHO is around 5.0 (15). The introduction of supplements rich in PUFA (80–82) prevents or minimizes biohydrogenation and affects the carcass characteristics (83). Albertí et al. (84) found that addition of 5% linseed decreased the dressing rate without changing the daily gain or classification of carcasses. Additionally, a lower n-6/n-3 fatty acid ratio has been observed in muscle from grass-fed animals compared to that in concentrate-fed animals (32).

Murphy et al. (85) reported that increased toe net growth does not adversely affect the walking ability. Despite the greater toe net growth in bulls accommodated on rubber flooring, there was no effect of floor type on locomotion score, suggesting that the increased toe net growth does not adversely affect the walking ability.

## Effect of Sex on Beef and Carcass Quality

The research carried out by Bureš and Barton (86) showed the impact of the sex and age of slaughtered animals on carcass composition, feed intake, growth, and quality of MLL meat on Simmental × Charolaise heifers and bulls. The results showed that the body weight of the bulls increased, while the daily dry matter consumption was higher. They obtained a significant interaction between sex × slaughter age and feed conversion ratio which decreased in older heifers. The bull carcasses were leaner with a higher total meat proportion. Bull carcasses obtained a higher proportion of the high-priced shoulder meat, and heifer carcasses had a better meat proportion of loin and rump. The proportion of bones and high-priced meat has decreased with the age of animals, whose carcasses were also fatter. Bulls had less dry matter, proteins, and intramuscular fat and more collagen than heifers.

Richardson and Herd (87) indicate differences between age and sex groups in terms of feed conversion ratio, which is caused by a few biological mechanisms, e.g., protein turnover, different body composition, or tissue metabolism of animals. The higher internal fat deposition in the heifer group determined a lower killing-out proportion. The same conclusions were reported by Steen (88), Frickh et al. (89), and Velik et al. (90). The fatness characteristics were affected by slaughter age and sex. Bulls had a lower proportion of fat in their body composition than heifers. Both groups produced more fat with increasing age; however, this trend was more intense for heifers.

The different meat distribution in bull and heifer carcasses showed a more intensive meat expansion in the forequarter in bulls and hindquarter in heifers. The respective research of Steen and Kilpatrick (91) and Link et al. (92) comparing the carcasses of different breeds of bulls and heifers were in agreement with the results of Bureš and Barton (86). With age, the proportions of high-value meat are decreasing, causing a fall of high/low-priced meat ratio, e.g., the MLL content per 100 kg of slaughter weight is smaller; the fat content is also higher with age. Harper and Pethick (93) documented the influence of sex hormones on intramuscular adipocyte development. This study showed almost twice higher intramuscular fat content in heifers (petroleum ether extract) than in bulls of the same age. The papers discussed have shown significant differences between both sexes slaughtered at two fixed ages in terms of performance, parameters of meat quality, and carcass traits. The heifers grew slower and less effectively, had a lower killing-out proportion, and produced fatter carcasses with a lower total meat proportion than the bulls. The MLL of bulls compared to heifers contained more intramuscular fat, less protein, less dry matter, and more total collagen, which was assessed by the sensory panel as more acceptable. The increase of slaughter age by 4 months resulted, especially in heifers, in reduced daily gain and feed conversion ratio as well as markedly higher fatness characteristics. Therefore, such an extension of the finishing period could not be considered advantageous for Charolaise × Simmental heifers fed a high-energy diet. Shifting the term of slaughter for 4 months caused a decrease of feed conversion and daily gains, especially in the heifer group. Therefore, application of high-energy diet in the finishing period was not efficient to Charolaise × Simmental heifers. Additionally, Prado et al. (94) reported that the finishing of young bulls in feedlot is to be recommended since the animals produce carcasses with higher amounts of edible meat and higher yields of commercial cuts, thus allowing for a better price for the carcass.

Many researchers (6, 95, 96) indicate sex as an important factor causing differences in meat quality. Additionally, heifer carcasses also have a higher fat/meat proportion (97, 98).

## Conclusion

There is a well-recognized impact of breed on lipid metabolism in tissues and dietary fatty acid content in bovine muscles. Based on the literature reviewed, it can be concluded that the quality of beef is largely related to sex, age of the slaughtered animals, and feeding system. All of these factors must be taken into consideration when addressing improvements to the nutritional quality of beef.

## Author Contributions

TS: contributed to conceptualization, searching of literature, writing—original draft, writing—review and editing, project administration, and supervision. KP: contributed to conceptualization, writing—original draft, and writing—review and editing. MG, JS, PK, and PS: contributed to searching of literature. GG: contributed to visualization. All authors read and approved the final manuscript.

## Funding

The authors acknowledge the financial support for this project provided by transnational funding bodies, being partners of the FP7 ERA-net project, CORE Organic Plus, and European Commission co-fund.

## Conflict of Interest

The authors declare that the research was conducted in the absence of any commercial or financial relationships that could be construed as a potential conflict of interest.

## Publisher's Note

All claims expressed in this article are solely those of the authors and do not necessarily represent those of their affiliated organizations, or those of the publisher, the editors and the reviewers. Any product that may be evaluated in this article, or claim that may be made by its manufacturer, is not guaranteed or endorsed by the publisher.

## Footnotes

^1^Dyrektywa Komisji 2001/101/WE z dnia 26 listopada 2001 r. zmieniajaca dyrektywe 2000/13/WE Parlamentu Europejskiego i Rady w sprawie zblizenia ustawodawstw Państw Członkowskich odnoszacych sie do etykietowania, p., Ed.
